# Improving public safety in events of mass gathering: The 2022 Kanjuruhan Stadium Disaster in Indonesia

**DOI:** 10.1002/puh2.139

**Published:** 2023-11-15

**Authors:** Lowilius Wiyono, Irfan Kresnadi, Awliya Syamsul Munir, Medhavini Tannuardi, Listya Tresnanti Mirtha

**Affiliations:** ^1^ Dr. Abdul Aziz General Hospital Singkawang Indonesia; ^2^ Faculty of Medicine Universitas Indonesia Jakarta Indonesia; ^3^ Sports Medicine Residency Training Program Department of Community Medicine Faculty of Medicine Universitas Indonesia Jakarta Indonesia; ^4^ Hospital of Universitas Indonesia Depok Indonesia

**Keywords:** disaster medicine, FIFA, football, Kanjuruhan, mass gathering, safety and security, sports mass gathering

## Abstract

The Kanjuruhan tragedy has caused tremendous sorrow for Indonesia and its football history. The incident happened during the match between the Arema and Persebaya football teams that was followed by a chaotic stampede due to supporters’ anarchy. The incident caused 135 deaths due to a stampede in the chaotic stadium. However, it has also brought into the limelight several violations and depravity of safety measures that have been neglected by the Indonesian officials and its football association. Despite being banned by FIFA's Safety and Security Regulations, the use of crowd‐control gas is hypothesized as one of the key driving factors for the stampede. There were also violations of the evacuation plan with overloaded stadium capacity, lack of safety protocol, and no regard for the disaster management command chain needed in such a situation. Inappropriate security plans, lack of safety officers, lack of a contingency plan in the event of a stampede, and lack of appropriate safe infrastructure contributed to the severity of this incident. We discuss the incident with the possible causes and notable issues in the management of mass gatherings, as well as the might‐be solution and precaution for all parties involved, from the evaluation of the use of crowd‐control gas, reassessment of safety protocol, and infrastructure to the appropriate response for stampede or any similar situations. The Kanjuruhan tragedy is heart‐wrenching, with various issues involved, showing the lack of regulations by the Indonesian government and its football association. However, it might also be an opportunity to learn and inspect all parties to achieve a peaceful and safe football in Indonesia.

## INTRODUCTION

On the night of October 1, 2022, after a football match between Arema and Persebaya ended in a 3–2 defeat for Arema at Kanjuruhan Stadium in Malang, East Java, approximately 3000 Arema's supporters entered the pitch in rage [1]. They swarmed the pitch in search of their team's players, wanting an explanation for the first defeat after 23 years of undefeated home matches against the Persebaya football team. Local police claimed that due to the anarchy the supporters were about to make, police officers had no alternative but to fire tear gas into the crowd [2]. FIFA's stadium safety guidelines forbid officials from using crowd‐control gas [3]. Witnesses reported that the gas was fired indiscriminately into the stands, prompting the crowd to panic and leave. Even though the stadium has 14 gates, some were locked or insufficient for evacuation [4]. In addition to the unavailable gates, there were no clear evacuation routes. This resulted in over‐capacitated crowds pressing against one another, causing a stampede where victims were trampled on and suffocated to death as they tried to evacuate through constrained or locked exits [5]. Aside from entering the pitch, the supporters were claimed to have destroyed 10 police cars and 3 private cars [6].

The reported number of victims varies between sources, but there were at least 754 victims, with 135 dead, making the patient presentation rate of 17.95 per 10,000 attendants in a single event [7]. The deaths at the stadium are the second‐deadliest in the history of football after at least 328 people were killed at the 1964 Estadio Nacional in Lima, Peru [10]. These events were also preceded by a crowd of people that the authorities could not control. Stampedes, as impulsive mass movement causing injury or deaths, in football matches have happened a few times in history, such as the Hillsborough Stadium incident in Sheffield with 97 deaths and 766 injuries, the Johannesburg Ellis Park Stadium incident in South Africa, or Abidjan's Felix Houphouet‐Boigny stadium incident in Ivory Coast. These incidents are caused by similar things, stampedes due to overcapacity, excessive influx of spectators, and poor crowd management [11, 12]. Therefore, the threat of stampedes should always be anticipated in mass gatherings or sports events.

Despite the nonbinding regulation of FIFA, The Indonesian Football Association (PSSI) also adopted FIFA's Stadium Safety and Security Regulations as a benchmark for its own safety regulations [13]. As the regulations were already available with safety and security measures being implemented during the match, how the Kanjuruhan tragedy occurred needs careful evaluation.

## SAFETY VIOLATIONS IN THE KANJURUHAN STADIUM

### Use of crowd‐control gas

The tragedy has revealed several violations, starting with the use of crowd‐control gas or 2‐chlorobenzalmalononitrile (agent CS), as the leading cause of the stampede as stated by the government [13]. Act 19 of the FIFA stadium safety and security regulations specifically states not to use firearms or crowd‐control gas to maintain public order on the stadium or field, which was adopted by the PSSI [3, 14]. Agent CS has been known to have negative health impacts, including dermatologic, respiratory, and ocular effects. Long‐term effects, such as hypersensitivity reaction and reactive airway dysfunction syndrome lasting 6 months to 2 years, occur in more than 30% up to 10 months after exposure. The use of agent CS also contributes to higher mortality due to difficulties in intubation due to immune response postexposure [15].

### Violations of emergency exits and evacuation plan

There were also violations regarding evacuation planning, such as the obstruction of emergency exits, exit gates, and other routes to allow an orderly exit during the stampede [3]. According to PSSI's Stadium Regulations (Act 21), all of the stadium gates and exits need to be equipped with locks that are easily operated by the safety officers [14]. The gates and exits also need to be open at all times when there are spectators inside the stadium. However, based on witnesses, gate 13 of the stadium was closed, contributing to the stampede and panic among the spectators. Arema FC claims that the gate has been opened 15 min prior to the end of the match, which is still in violation of the regulation [16]. Although the number of exits is not regulated by the guidebooks, only the turnstiles have their own measurement (660 seats per turnstile). Despite the clear regulation by FIFA on turnstiles and the use of emergency exits, the PSSI regulations lack clear instructions regarding the exit plan.

### Lack of safety officers and safety protocol

According to FIFA's Stadium Safety and Security Regulations, the safety and security management of a football match is the responsibility of the safety and security management team, headed by a stadium security officer and a senior local police commander. The regulation also mentions crowd management, which highlights overcrowding, uncontrolled admission, and limited/non‐opening of additional entrances, as reasons for uncontrolled crowds [3]. These emergency plans should be communicated to local officials, firefighters, medical officers, police, and executive committees to prepare a swift and comprehensive plan [14].

**FIGURE 1:**
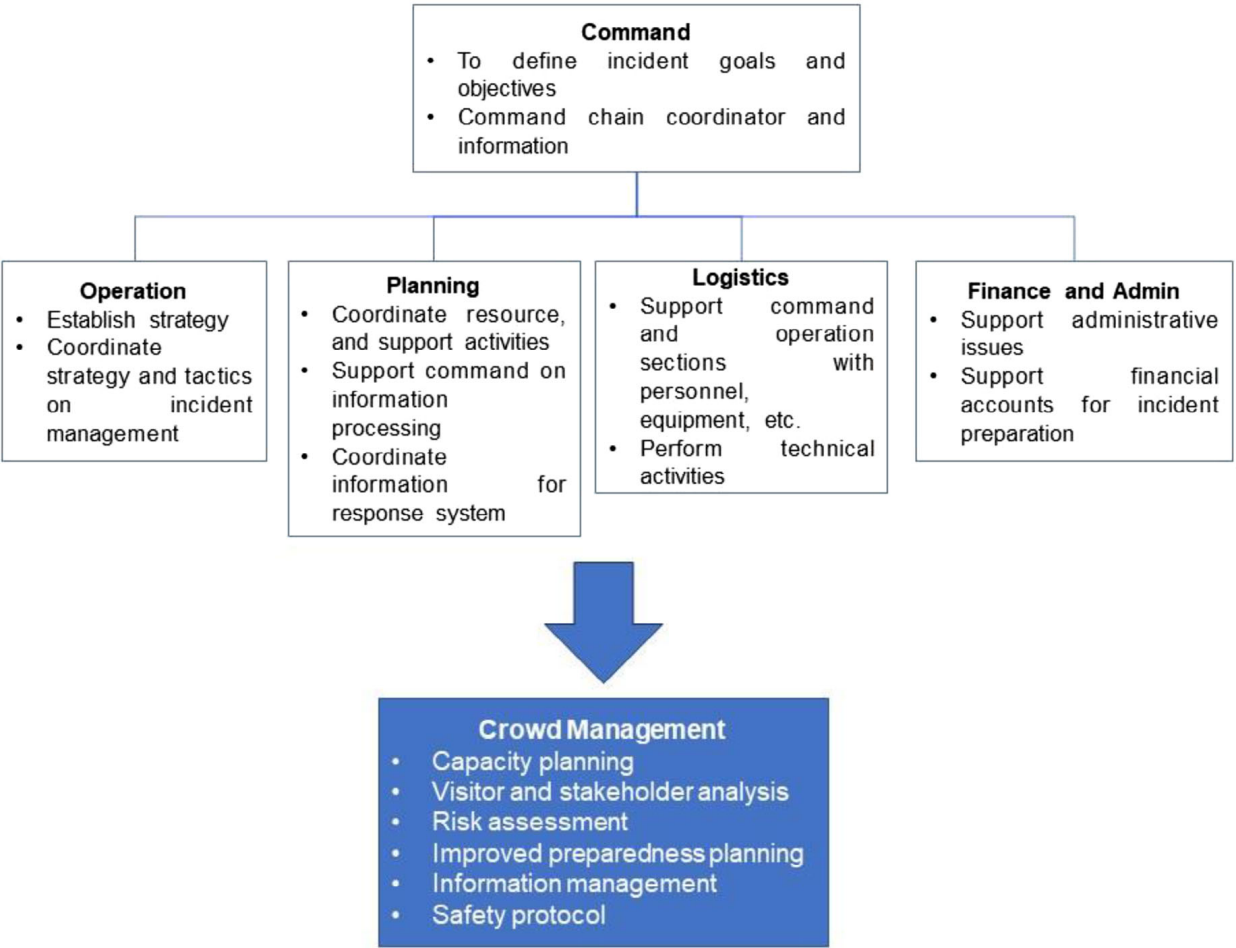
The incident command chain, which consists of command, operation, planning, logistics, and finance, plays a role in crowd management in identifying the needed elements of crowd management, particularly in stampede incident prevention.

The disaster management incident chain of command system is a guide for organizing the resources (system description) to respond to an incident and the processes (operational concepts). It is a standardized approach to onsite incident management leadership, control, and coordination that provides a common hierarchy within which personnel from multiple organizations can work effectively. It is about resolving “confusion about authority and responsibility” and reducing “resource scarcity and misallocation of existing resources” (Figure 1) [17, 18]. The incident command chain has set a goal in preventing such incidents with five parameters: understanding visitors and stakeholders, risk analysis and preparedness, information management and dissemination, safety and security protocol, and transportation and traffic management [19].

In the Kanjuruhan incident, the unclear chain of command structure might have played a role. This was an unclear chain of command for responders in the field resulting in a clear violation of safety and security protocols, the use of crowd‐control gas, delay in gate opening, and lack of a means to provide information or safety instructions to the spectators [20]. Insufficient understanding of the visitors and their behavior despite a history of fanaticism in each respective football club contributed a lack of preparedness to respond to such mishaps. The PSSI put the blame for this incident on Arema FC which was subsequently shifted to the league coordinator (Liga Indonesia Baru) [21]. According to FIFA's regulation, the number of security officers required is 1 per 250 spectators [3]. Although the match was guarded by about 2034 police officers and staff, they might not have been in the right positions/locations [22].

According to Act 21 of the FIFA regulations on stewards or safety officers training, the safety officers should be trained with roles or responsibilities, crowd management, emergency first aid, and contingency plans, but these are not clearly stated in PSSI and Liga Indonesia Baru (Indonesian football league) regulation [3, 14]. It is apparent that there may not have been optimal training of the safety officers who were in charge on that day. There was only one safety officer who fulfilled FIFA's credentials on that day [27].

### Overloaded stadium capacity

The number of spectators overloaded the stadium, with a higher number of tickets sold contrary to the actual capacity of the stadium itself. There were 42,558 tickets sold in the match. However, the actual stadium capacity is only about 36,222, contrary to the data provided by the local sports and youth authority, stating that the capacity is about 42,449 [8].

The physical structure of the stadium does not meet the FIFA stadium standards, except for the pitch, though official data on the stadium blueprints are quite hard to find. The tribunes, various supporting facilities, player and manager facilities, and proper signages are not available in the stadium, making it prone to nonoptimal contingency plans, particularly amidst the chaos during the incident [23]. Similar to other countries, this tragedy also highlights inadequate literacy on safety and security protocols by the government and league officials that regulate football associations and gatherings.

## LESSONS FOR PUBLIC SAFETY IN MASS GATHERINGS

This event serves as an opportunity to learn and prepare the country for safer and more secure sports events, for both players and spectators. The need for infrastructure reassessment with revision of the existing guidelines is essential. Regulatory bodies must ensure that such safety protocols for visitors and spectators are implemented.

### Infrastructure reassessment

A review of the public infrastructure must be done, with an emphasis on sporting venues capable of mitigating the effects of emergency scenarios. With Kanjuruhan Stadium failing to adhere to FIFA stadium standards, the government has planned to rebuild the stadium after a meeting between the Indonesian president and FIFA [25]. Reassessment of all stadiums should also be conducted, particularly on the facilities and the needed safety facilities.

The FIFA stadium guidelines should thoroughly be enforced, especially in holding international matches. Stadiums must have proper entry and exit gates suitable for mass‐gatherings, proper fences and walls on the perimeter of the stadium, emergency exit availability during the match, easy access for transportation particularly for the playing teams, and proper lighting with a minimal of 2400 lx. At present, there are only 14 stadiums in Indonesia that abide by the FIFA standard [26]. It is also important that there is proper management and maintenance of football stadiums.

### Safety and security protocol revision

The regulation on visitor safety should also be enforced as mandated by the law. Strict regulations on stadium capacity, safety protocols, and proper decorum should be enforced to prevent chaos and conflict among spectators. The need for officials to account for the fanaticism and culture of spectators and football club supporters has become necessary in making a risk assessment for such mishaps. The safety officer should also be aware of safety precautions to take in the event of a tragedy. More consideration needs to be given in choosing the appropriate number of safety officers and their deployment [3]. The safety officers should be available in various posts, such as crowd monitoring points, turnstiles, perimeter gates, restricted zones, and other zones, based on the initial risk assessment.

Strict security protocols also need to be conducted by officials and police officers. Due to the past nature of football fans in Indonesia, officials and league coordinators can separate the spectators or fans of opposing teams into different zones to reduce tension in the stadium. The use of “soft policing” could also be conducted by the police by making a peaceful barrier around the pitch nearing the end of the match. Police might also use other attributes such as hoodies or shirts instead of riot gear to soften the crowd response and reduce panic in the process [28]. The need for a clear evacuation plan, emergency informing, and coordination for crowd dispersal to every exit gate for effective crowd management should be further regulated by both FIFA and PSSI safety and security regulations [28]. The meeting between FIFA and the Indonesian government also states the need for cooperation and joint effort in formulating security protocols and procedures by both parties to prevent similar incidents.

### Crowd‐control measures and medical support

The emergency response committee can consider options for crowd control such as physical barriers to fragment the crowds. It is inappropriate to use crowd‐control gas, particularly in an enclosed space. Emergency medical staff and appropriate medical supplies should be prepared beforehand [30].

### Awareness on safety protocols

Spectators should be aware of standard safety protocols for crowds in similar situations. Organizers should educate visitors through educational videos or clear signs in stadiums [31]. Information on safety measurements, evacuation plans or pathways, instructions on the use of various safety facilities, and other instructions to support safety in the crowd should be a priority.

### The Indonesian government's plan of action

Despite almost a year of the process after the incident, a concrete evaluation and revision of safety protocols in the Indonesian football league system has not been discussed [32]. The government focused on the renovation of the Kanjuruhan football stadium based on the FIFA stadium standard. The people also heavily criticized this renovation plan and claimed the government to purposely hinder the legal process ensuing this case.

## CONCLUSION

The Kanjuruhan tragedy is a scar in the football history for Indonesia and the world. The incident has brought into the limelight various violations of standards and negligence of safety measures leading to a loss of lives. There should be a serious enforcement of regulations and laws, improvement of medical and safety protocols, and an improved awareness of officials and the public on safety measures in mass gatherings. This event serves as a painful lesson to ensure that public gatherings for sports, religious gatherings, and political rallies happen in a safe environment.

## AUTHOR CONTRIBUTIONS

*Conceptualization; writing—original draft*: Lowilius Wiyono. *Conceptualization; writing—original draft*: Irfan Kresnadi. *Conceptualization; writing—original draft*: Awliya Syamsul Munir. *Conceptualization; writing—review and editing*: Listya Tresnanti Mirtha.

## CONFLICT OF INTEREST STATEMENT

There are no conflicts of interest to be disclosed on the making of this manuscript.

## FUNDING INFORMATION

The authors declare no funding or sponsorship awarded in the making of this manuscript.

## Data Availability

The data in this manuscript is not available as there is no data synthesis or dataset used in this manuscript.
